# #Plugging Into Hope

**DOI:** 10.1177/1077800420960186

**Published:** 2021-09

**Authors:** Esther Fitzpatrick

**Affiliations:** 1 The University of Auckland, New Zealand

**Keywords:** plugging in, hope, art-making, interconnected, ontology

## Abstract

This is a critical autoethnography of my “plugging into hope” through engagement with the Massive_Micro project as I was, literally and simultaneously, plugging into my computer during the COVID-19 pandemic isolation. In this article, I demonstrate the art of #plugging into hope. Through drawing on a range of visual, poetic, and narrative data generated throughout this project, I write my story, creating an assemblage of plugging in moments with human and non-human. Adding to an ongoing conversation with art-making, writing, and my scholarly ghosts. Highlighting the significance of deliberately plugging into embodied intra-action with our world/s—connecting to hope.

I watched the recorded conversation between Anne and Annette on Zoom; recorded, edited, and shared with all of us, about where to go next with the Massive_Micro project. Annette asked about the so-whatness of the project. I paused and pondered, typed onto my word doc: #Plugging into hope. Then took some time to locate my original abstract, not remembering much what I had said, or why I had “plugged in.” Finally, I located the original and it all made sense: “Something to look forward to, hope redefined in COVID 19 through *being with.*”

This is a critical autoethnographic account of my “plugging into hope” through engagement with the Massive_Micro project (Markham & Harris, 2020) as I was, literally and simultaneously, plugging into my computer during the COVID-19 pandemic lockdown—and I still am plugging into hope. Through sharing some of my small stories of intra-action with the non-human and human (Barad, 2007; Haraway, 1997), and encouraged by the prompts, I demonstrate the importance of “making” and being “creative,” and of deliberately plugging into these moments. Roder (2011) describes intra-active pedagogy/method where learning is seen to take place in an ongoing material-discursive flow of agency (p. 61). Barad (2003) describes discourse as not *what is said* but rather “it is that which constrains and enables what can be said . . . [a] field of possibilities . . . not static or singular but . . . a dynamic and contingent multiplicity” (p. 819). So too, our actions throughout the Massive_Micro project were intra-actions; not the result of our individual “supreme” agency but the outcomes of our actions and the actions of others—human and non-human beings and materials (Barad, 2010; Haraway, 1997). I write this as the noise of a screeching vacuum cleaner and a tumbling drier clash with my every thought. I want to unplug those plugs! Such are the tensions of our world. We plug into the daily grind of what we have to do to survive, and plug into art-making to make sense of the daily grind.

## In This Paper I Demonstrate the Art of #Plugging Into Hope

Through drawing on a range of visual, poetic, and narrative data generated throughout the Massive_Micro project, I write my story, creating a research assemblage of plugging-in moments. An assemblage comprised bodies, things, and abstractions “caught up in this social inquiry, including the events . . . the tools, models and precepts of the research, and [me] the researcher . . .” (Fox & Alldred, 2015, p. 400). This story is my performance of deliberately plugging into creative making, into theory, into writing, to create a “living bod[y] of thought” (Holman Jones, 2016, p. 8). It is not my story alone. On Facebook a friend’s daughter asked “what have I got to look forward to?” Outside my room it’s raining. Through an open door a gentle breeze tickles my neck. Big, fat raindrops plop. I imagine rain on the ancient Kōwhai tree, trickling down its moss heavy arms, carrying stories of my place before/since I arrived. In our New Zealand bubbles we still ourselves, slow down and attune ourselves, to our bodies, our interactions with the world, listening more to that breeze on our neck. This strange quiet bubble I now live in, a corner of the world at the edges of our map, where I am both engaging with the wind, the rain, and my forever-sitting-plugged-into-machine aching body, while also intensely engaged with Facebook posts of a friend finding a puppy for her daughter, thousands of miles away from me—so she may have something to look forward to.

I write small stories with my scholarly ghosts, who always question what I do and add to/edit/inspire my stories. These ghosts are in “sympathy” with my writing, my always-present-absent collaborators (St. Pierre, 2014). St. Pierre speaks of “one writing machine plugging into others—the work of the writer writing” (p. 376). This ongoing conversation with art-making, writing, and my ghosts relates to what Jackson and Mazzei (2012) describe as “reading-the-data-while-thinking-the-theory as a moment of plugging in, of entering the assemblage, of making new connectives” (p. 4). The Massive_Micro project produced an assemblage of author—text—world, *although never linear*, through deliberate intra-actions with the non-human and human worlds of our bubble, through the act of making, through the collaborations and sharing across the digital spaces, and through the writing. As Holman Jones (2016) argues “the insights of theory—including queer theory—only become useful to us when they are presented in context, in practice, and performance, in people’s lives” (p. 5).

## I Deliberately Play Here With the Term “Plugging in”

The process of “plugging in” originates from Deleuze and Guattari (1983) who ask

A book itself is a little machine; . . . We have been criticized for overquoting literary authors. But when one writes, the only question is which other machine the literary machine can be plugged into, must be plugged into in order to work. (cited in St. Pierre, 2014, p. 376)

Elsewhere I have argued about the importance of the arts-based researcher as “serendipiter” (Fitzpatrick, 2017), one who deliberately notices, who plugs themselves into arts-based methods, making sense of the issue/pondering/world through an embodied response. Here I also contend, as critical autoethnographers, we need to plug into theory to make deeper and more critical connections between our micro lived stories and the macro wider social, political, historical, and environmental factors that are shaping our worlds/stories. It is a process of “[p]lugging in to produce something new . . . a constant, continuous process of making and unmaking. An assemblage isn’t a thing—it is the process of making and unmaking the thing” (Jackson & Mazzei, 2012, p. 1).

## An Assemblage of Plugging in Moments

Throughout the 21 days of the Massive_Micro project, my deliberately posting on Facebook what I was thinking/creating/making/doing enabled me to push/plug myself into interaction and/or collaboration. I argue the simple act of “plugging into the project” provided me with an excuse to *unplug* myself from other demands. I am reminded of Haraway who argued “[o]ur machines are disturbingly lively, and we ourselves frighteningly inert” (Haraway, 1991, p. 152).

We need ways to plug into hope.

### FB Post

**Figure 1.**
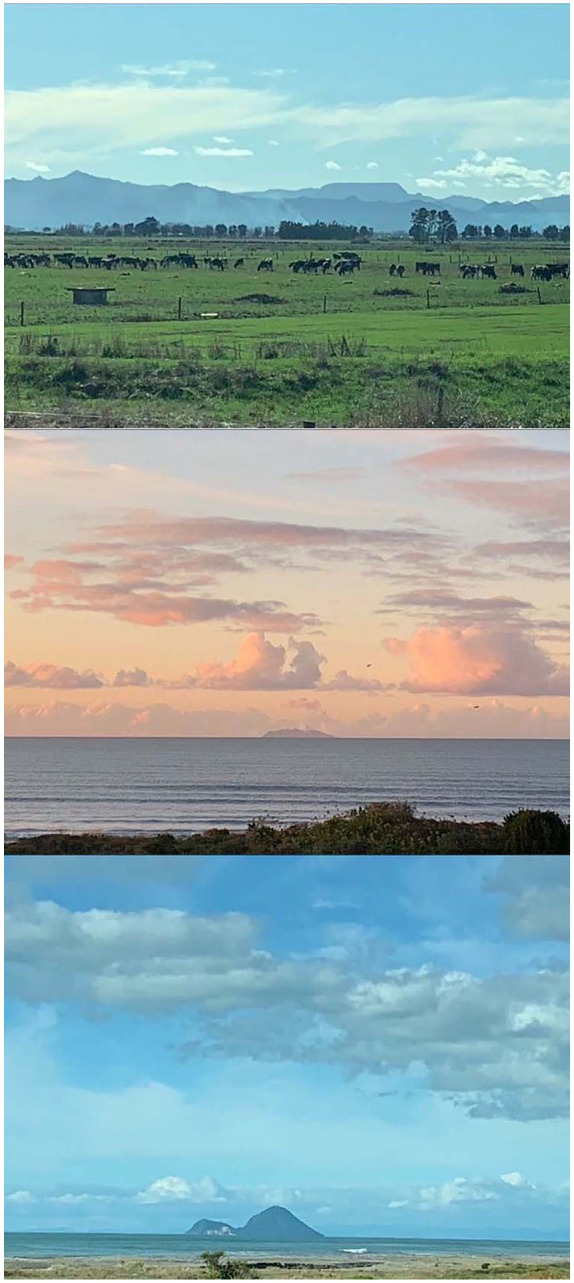
“Ūkaipo” photos by author.

Kia ora from Aotearoa New Zealand—now at Level 2. I have driven home today—to the place I call my Ūkaipo (Figure 1)—way down the East Coast. Ūkaipo—place of contentment in Indigenous Māori language (see Fitzpatrick, 2018). Although I am Pākehā, a descendent of settler ancestors—this is the place I was born, my home.

I plug myself into the sensory experience. Autumn, vivid and dancing with a blue sky backdrop. I drank her in. I zoom past mountains, a hawk flies low showing off feathery britches, I laugh at his performance. Whakaari (our volcanic island) puffed out billows of white smoke and I made her promise to behave. She is known for her temper tantrums. Eventually the long blue coastline paraded before me, my heart sang. Back home here for 1 week where I can hug my mum-in-law, plug into a Zoom meeting, review articles, . . . well everything I need to in my role as a lecturer for the Education Faculty at Auckland. I also get to “pull the plug” on my computer and become plugged into talking to/with my beach, engage with my ocean, dig my toes deep into the sand I know, and drink up the scent of this place. I truly am home.

### Reflective Note

My going home is/always a deeply embodied experience, involving entangled ontologies, hauntings, embodied ethnicities (see Dion et al., 2011; Fitzpatrick, 2014, 2018). A Māori world view understands “all objects, ideas, places, animals, forces and people are in ‘full relationship’ and therefore influence each other” (Mika, 2017, p. 123). Everything in existence is meaningfully interconnected. Or as Stewart-Harawira (2013) argues, “[f]rom an Indigenous perspective, everything is living. This includes inanimate objects that are understood to hold their own energy . . . are connected to the energetic web of the entire planet” (p. 44). Māori scholar Te kawehau Hoskins (Jones & Hoskins, 2016) explores the work of new materialist theories by considering the always/ongoing Indigenous Māori worldview and describes our relationship with “things,” and importantly our environments, “as determining events, as exerting forces, as volitional, as instructing people, as speaking to us—and our being able to hear” (p. 41). As a non-Indigenous researcher my identity too, as Pākehā, is a dynamic becoming in relation to my embodied experiences in a range of cultural environments. A fluid process of encountering, engaging, and entanglement with human and non-human. The liveliness of things, vividness, as new materialist writer Jane Bennett (2010) argues, is significant to how I understand my active engagement with materiality and bodies.

**Prompt #8**: How can one frame feel like the whole world? . . . make a page of comics . . .

### Reflective Note

**Figure 2.**
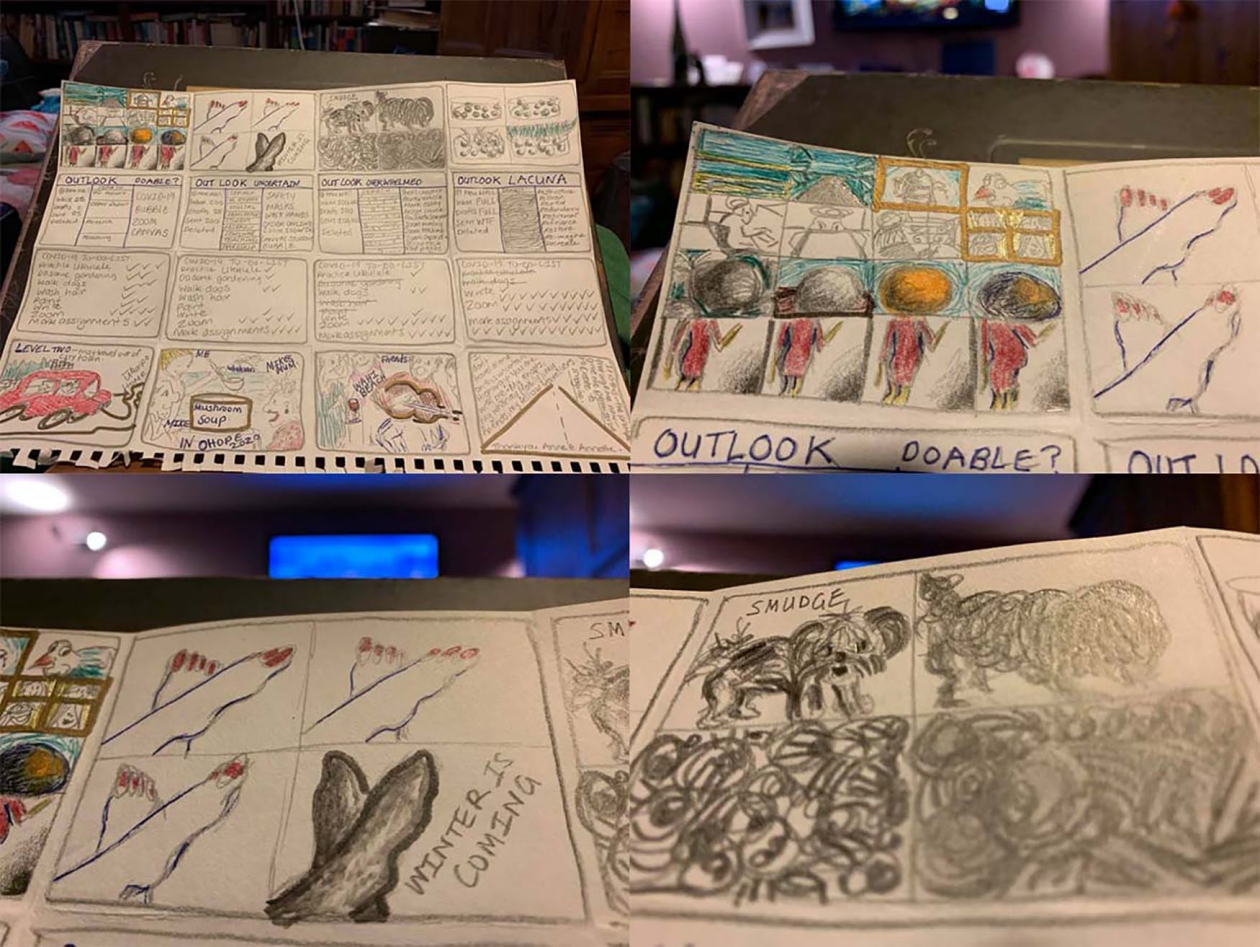
“A story inside a story” artwork by author.

**Figure 3.**
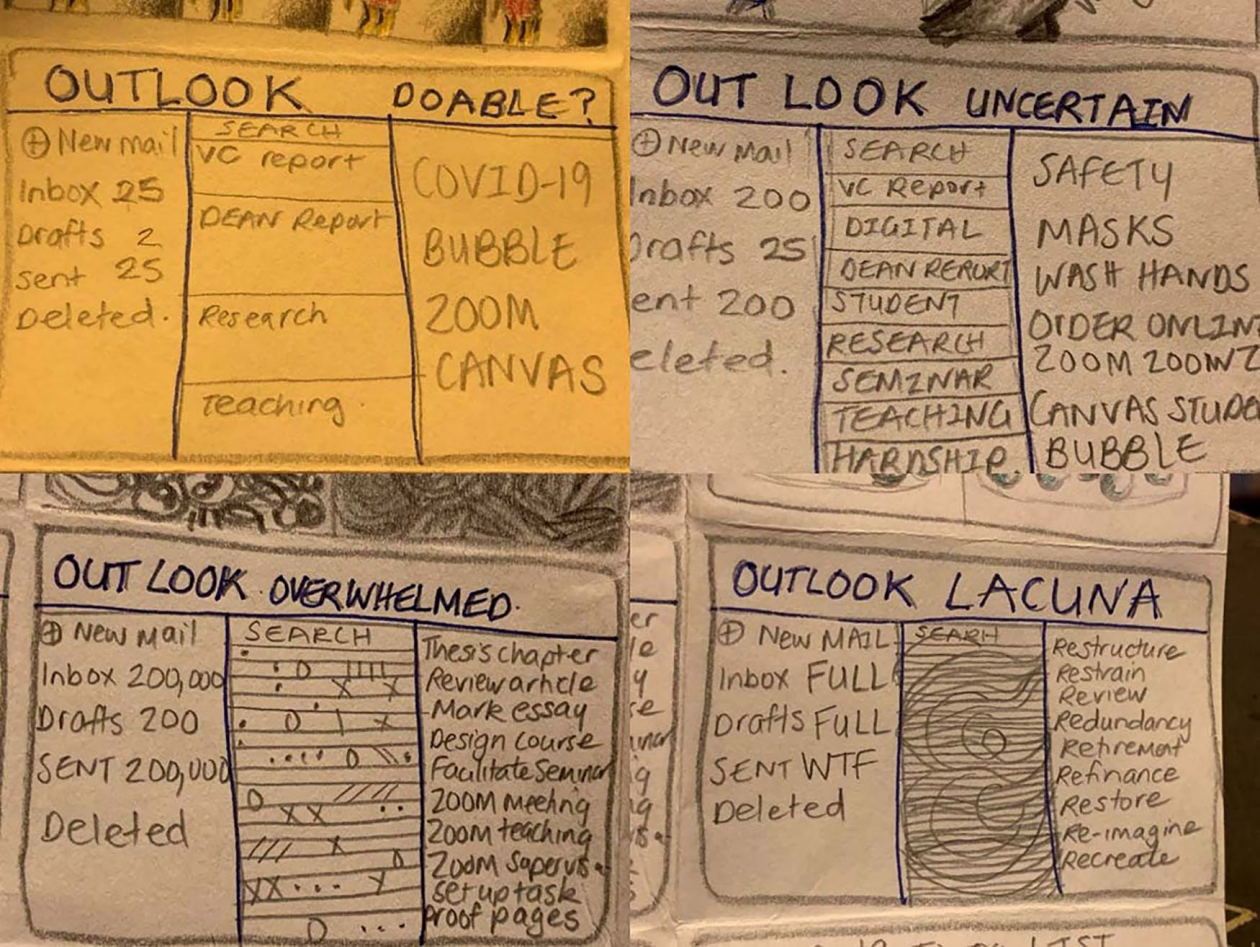
“Outlook changes” artwork by author.

I hesitated, then began drawing the square(ish)s, then squares inside squares . . . stories inside stories (Figure 1) . . . worlds inside worlds . . . metafiction, intertextuality (Figure 2) and (Figure 3) . . .

Annette Markham (2008) describes her coming face-to-face with privilege when, after moving to a new home, was unable to plug directly into a trustworthy electric power supply and/or strong reliable internet connections. She writes how her

everyday behaviors were developed in a cultural context of ready access to basic goods and services, [her] modes of communication were overly dependent on electronic technologies, and [her] working theories about new technologies for communication were embedded in invisible infrastructures of privilege. (p. 132)

I too ironically was privileged through this time, being able to plug into a reliable power source with constant strong internet connection, and skilled support people. Rereading Donna Haraway’s (1991) “A Cyborg Manisfesto,” I feel akin to the Cyborg. As I plug in daily to my digital devices, plug them into the power socket, plug posts of my bubble life onto facebook, “the possibility open[ing] up for weaving something other than a shroud for the day after the apocalypse” [I hope] (Haraway, 1991, p. 156).

**Figure 4.**
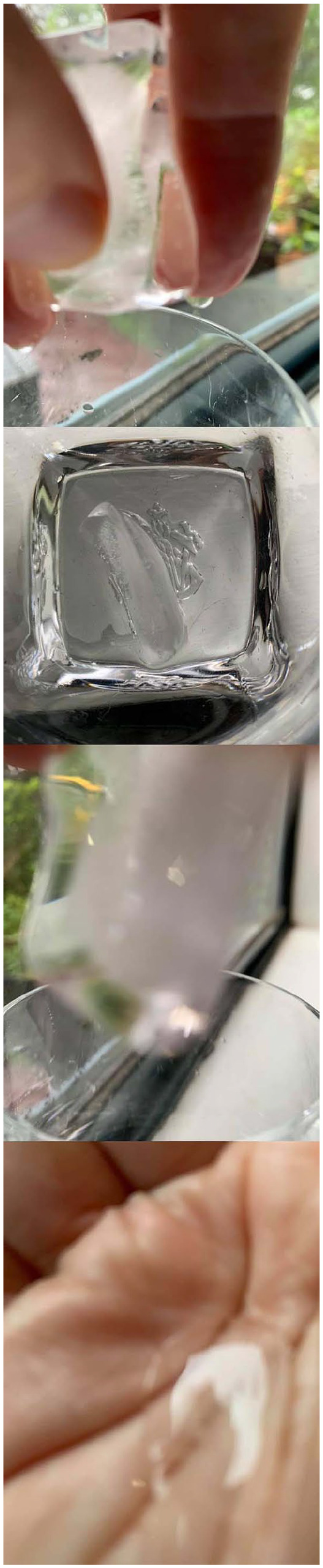
“Ice and me” photos by author.

**Prompt #9:** I really didn’t want to *do nothing* while an ice cube melted in my hand (Figure 4). But of course there was no nothing, it was really quite something.

## ICE CUBE Story 2: A Found Poem

**The gap I didn’t even know existed.**

Ice burning the palm of my wrinkly hand.

Swimming, floating, *skin on my skin.*

 (I don’t have time for this!)

Dripping into a Whiskey glass, a

Vessel worthy of a cube of ice.

**Ancient renewed relationship of.**

Glass and water, *stable and unstable*, liquid and solid,

**In constant movement.**

Ice morph[s] into liquid.

Joy replacing frustration I begin . . . *to play*.

Wrangling the cube in palm, whiskey glass on . . .

Window ledge . . . *Catching ourselves* in the camera lens

Slippery ice . . . catch[ing] the *dying light of a grey damp day*

Edging toward late.

**Together we were dancing.**

Laughing . . . sometimes at

A pair of tweezers with several fleshy prongs,

*alive and morphing*,

**Did/didn’t want to be picked up.**

Twisted and turned.

Catching the trees on the edge of the bush,

Grey wet sky, dirty window, recycling bin with its yellow lid,

Edges of the glass, bottom of the glass.

Becoming accustomed to my hand.

I surrender.

**My hand . . . belonging with ice.**

Settling into the crevices of the creases,

Mapping out my lifeline,

**We drip together,**

Puddled in my palm [you] slipped through my fingers

Not wanting to *lose you as I lost you*,

**As I am still losing you.**

A memory of a long ago writing

Glaciers and rivers, and streams, and steam,

Seas and clouds and snow, hail and rain.

For a degree in environmental geography . . .

**I must let you go again.**

Through plugging into noticing, *being with* my intimate/familiar objects, a material-discursive practice, taking photographic images, and writing, my ongoing entangled relationship with “things” was illuminated. As Barad (2003) contends,

*[m]aterial-discursive practices are specific iterative enactments—agential intra-actions—through which matter is differentially engaged and articulated* (*in the emergence of boundaries and meanings*), *reconfiguring the material-discursive field of possibilities in the iterative dynamics of intra-activity that is agency*. (p. 823, italics in the original)

The holding, being with an ice cube as it melted, the intra-action/s, a causally constraining nondeterministic enactment, through which matter-in-the-process-of-becoming was sedimented out and enfolded in further materializations (Barad, 2003, p. 823).

**Prompt #11:** Victorian Allegorical maps of COVID. In Victorian times, it was popular to draw maps of the sentiments, or allegorical maps. Using this sensibility, draw a map of yourself. Use color, be playful. Over the next few days, build onto this map, or create different variations of this map.

**Figure 5.**
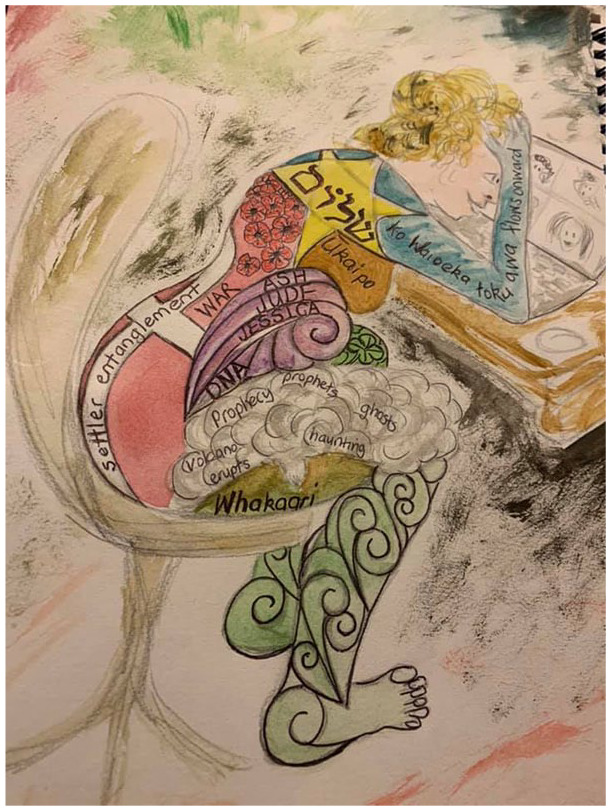
“An ongoing entanglement with DNA, rivers, mountains, language, family, the world of play, the world of work, survival, hope.” *Note.* Artwork by author.

For Ingold (2013), in making something we are engaged in “transformational” rather than “documenting” learning (pp. 2–8). Arts-based research then provides a critically reflective way for me as researcher to engage in a material and creative activity, to explore and imagine future possibilities, through reimagining our histories (see Figure 5).

Back to Haraway (1991) and the imagery of cyborgs . . . “[o]ur bodies, ourselves; bodies are maps of power and identity. Cyborgs are no exception” (p. 179). I am a cyborg becoming.

**Figure 6.**
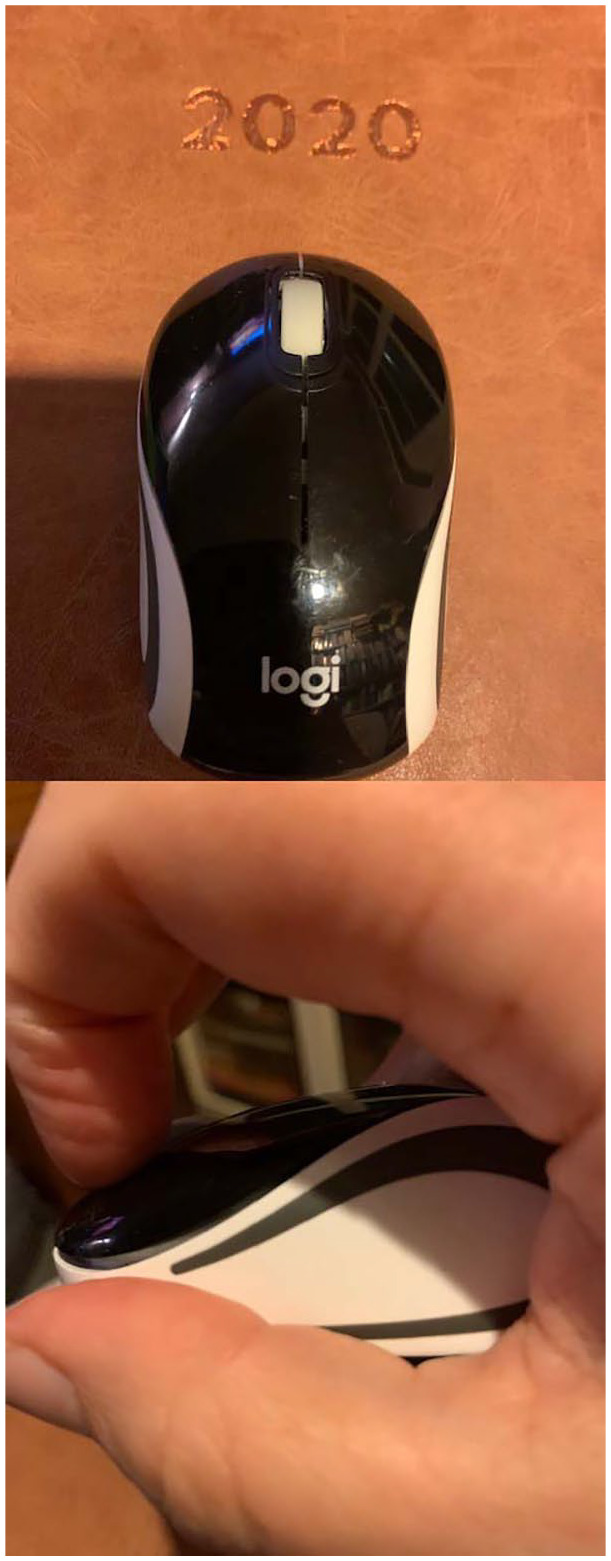
“Mouse and me” photos by author.

**Prompt #10:** In your field diary today, think about machines as agentic, technology as relational, automation as mediators (Figure 6).

I read the prompt after a day of Zooming—with my hand clasped painfully around your smooth light frame . . . you being temperamental—again.

*Come on*, I’m half way through this Zoom meeting, they’re all there, *look*, waiting for me.

We have a time slot, scheduled.

At the right hand corner of the screen is a reminder, popping up like hot toast, shouting out “eat

me”! You are sluggish,

did I work you too hard yesterday?

You don’t like the way I grab and thrust?

*Aren’t you my machine?*

Do I need to pay homage to your delicate nature,

grant you power, stroke your ego, release you.

I need you to behave, to fall into agreement, I have set in this agenda.

They are waiting, she is tearful, things are/have not been going to plan.

No—I can’t rest you.

You are my hope of connection, my hand and you in unison,

My relationship with the computer, with the mouse, in connection with the computer, in connection with worlds of work, of other bubbles, of politics, of COVID-19 happenings.

My body as cyborg “is not innocent.”

As Haraway (1991) suggests,

[i]ntense pleasure in skill, machine skill, ceases to be a sin, but an aspect of embodiment. The machine is not an it to be animated, worshipped, and dominated. The machine is us, our processes, an aspect of our embodiment. . . . We are responsible for boundaries; we are they. (p. 179)

**Plugging into performative writing** through engaging deliberately and creatively with the human and non-human world is something I am keenly interested in as pedagogy and methodology (see Fitzpatrick & Longley, 2020). The Massive_Micro project enabled me to focus on the *performance* of writing, as bodies in intra-action through embodied, felt, sensory methods in response to my experiences in my bubble. *Performative writing*, a method of “writing as doing” where “writing becomes meaningful in the material, a dis/continuous act of writing” (Pollock, 1998, p. 75). Ellis describes the creative process as a confluence between forces and materials, to “[soften] distinctions between organism and artifact” (Ellis, 2015, p. 98). If material and researcher “in their reciprocal, intra-active entanglement, are not fixed conditions but rather emerging possibilities” (Battista, 2012, p. 72), what (knowledge, documentation, relationship, identity . . .) might manifest from such entanglement? How might the affective forces of ecologies, spaces, and creative processes morph together?

**Plugging into making and writing** with the Massive_Micro prompts provided me with hope. During the experience and engagement with the 21 prompts, I was mostly plugging in daily to an increased workload of responding to anxious students and colleagues. Literally we (and others globally) transferred our face-to-face teaching, supervision, and service responsibilities onto a digital platform and plugged into Zoom, Google-docs, emails, Facebook messenger, and other platforms. Weekends became a thing of the past. The equity divide became more apparent. The privilege of still having a job, although with increasing workloads, plus the precarity of the job situation, wreaked havoc on our sensibilities—and from New Zealand the strangeness of it all was illuminated daily on Facebook and via Zoom meetings with international colleagues, family, and friends. In the midst of all this someone close to me asked why I was doing this project when I wasn’t coping with my workload. I replied, “this is something I find joy in.” Unplugging myself from Zoom and plugging into making, creating, after a long day. Reaching up to find the cane basket, wiping the dust off my pens and pencils, and finding an old art journal. Being brave, playing with the ideas, writing through the ideas (Richardson & St. Pierre, 2005), sharing the ideas—pushing myself to engage and to collaborate. *Doing* the theory. Remembering that . . . The idea that disaster will come is not new; . . . resurgence of peoples and of places is nurtured with ragged vitality in the teeth of such loss, mourning, memory, resilience, reinvention of what it means to be . . ., refusal to deny irreversible destruction, and refusal to disengage from living and dying well in pasts, presents, and futures (Haraway, 2019, p. 569).

### Reflective Note

**Figure 7.**
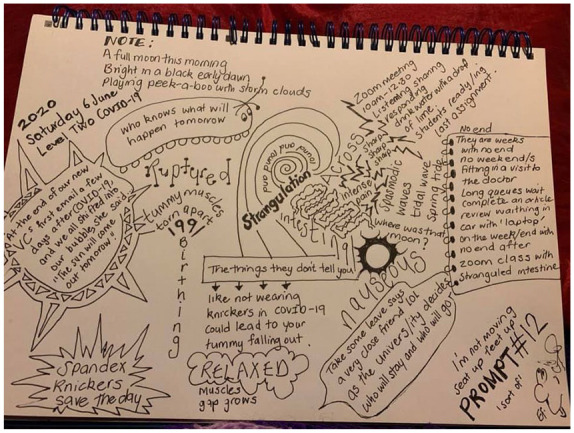
Prompt #12.

Prompt #12 (Figure 7) was created the day I woke up with a strangulated hernia. Since then, New Zealand has shifted from level four back down to level one and we are now back at level three. Our university is closed, Auckland is closed, and we are back inside our bubbles. Since then I have had two operations, my dad has been in ICU, and my daughter has given birth to our first grandchild. The workload only steadily increases and the stress in our communities is huge. This morning a friend, New Zealand poet Karlo Mila, shared a poem on Facebook titled “Travelling”—about our digital connections while in isolation. Being with the Massive_Micro project provided a way to make sense of what Mila describes as being “hard-wired for connection.” “So, what next” whispers Annette Markham (2008) as she cooks toast and covers it with crunchy peanut butter. I read her words and go downstairs to cook toast and brew coffee.

### #PluggingintoHope

Like a persistent nagging at our head/heart. Plugging into hope, into other ways of being, into other life forces, a pulse not our own, becoming us. Connection to hope. Connecting others to hope. Plugging into conversations, to ways of making, being, “kick-start” our heart beat again, and again. Plugging into the cry of a newborn baby in the room next door. Plugging into the sound of the Tūi outside my window, singing to the sunrise. Plugging into the soft breathing of the small black dog. Plugging into the waft and penetrating scent of freshly brewed coffee—even after the cup is empty. Even as I plug the computer into the power socket, as I scroll down, click, shift, down, past the newsfeed of hopelessness, plugging into hope, again far away with scenes so foreign like a Sci Fi movie that just won’t stop playing, deliberately plugging into making, to laughing with friends, to walking a newborn baby.
